# Preparation and Peculiar Magnetic Properties at Low Temperatures of La_1.85_Sr_0.15_CuO_4_ Nanofibers

**DOI:** 10.3390/nano14040361

**Published:** 2024-02-15

**Authors:** Shi-Long Gao, Ting-Ting Zhang, Li-Peng Qiu, Yu-Rui Zhang, Guo-Ting Cheng, Qi Liu, Wen-Peng Han, Seeram Ramakrishna, Yun-Ze Long

**Affiliations:** 1 Collaborative Innovation Center for Nanomaterials & Devices, Innovation Institute for Advanced Nanofibers, College of Physics, Qingdao University, Qingdao 266071, Chinahan_wenpeng@163.com (W.-P.H.); 2 Department of Electrical and Computer Engineering, Herbert Wertheim College of Engineering, University of Florida, Gainesville, FL 32608, USA; guoting.cheng@ufl.edu; 3 Center for Nanotechnology & Sustainability, Department of Mechanical Engineering, College of Design and Engineering, National University of Singapore, Singapore 117574, Singapore; seeram@nus.edu.sg

**Keywords:** superconducting, nanofibers, La_1.85_Sr_0.15_CuO_4_, electrospinning, ferromagnetism

## Abstract

Herein, the preparation process, morphology, structure, and magnetic properties of La_1.85_Sr_0.15_CuO_4_ (LSCO) cobweb-like nanofibers are reported. LSCO nanofibers with a regular grain size distribution are successfully prepared via electrospinning, followed by calcination. We conducted morphology analysis and elemental distribution using electron microscopy and energy-dispersive X-ray spectroscopy (EDS), respectively. Additionally, magnetic property testing was performed using a vibrating sample magnetometer (VSM) to confirm the superconducting properties of the samples. Interestingly, our samples exhibited a superconducting transition temperature, Tc, of 25.21 K, which showed some disparity compared to similar works. Furthermore, we observed a ferromagnetic response at low temperatures in the superconducting nanofibers. We attribute these phenomena to the effects generated by surface states of nanoscale superconducting materials.

## 1. Introduction

The revelation of superconductivity has ignited a fervent pursuit to unravel avenues for elevating the superconducting transition temperature. Metal oxides, renowned for their propensity to exhibit a superconducting state at low temperatures, have emerged as focal points of extensive inquiry [1,2,3,4,5]. A pivotal milestone in 1986 saw the lanthanum–barium–copper oxide compound achieve a superconducting transition temperature of 35 K, surpassing the theoretical threshold envisaged by the Bardeen–Cooper–Schrieffer (BCS) theory at 30 K [6,7]. This seminal breakthrough catalyzed a surge in research dedicated to copper oxide-based high-temperature superconductors [8,9].

La_1.85_Sr_0.15_CuO_4_ (LSCO), a pioneering high-temperature superconductor, has been earmarked for myriad applications. However, a litany of challenges, including poor current transport density, elevated losses, brittleness, and exorbitant production costs, impede its widespread adoption [10,11,12,13].

Superconducting nanofibers have emerged as compelling subjects of investigation in both fundamental research and practical applications owing to their distinctive wide transition temperatures, exceptional thermal conductivity, and high critical current density, among other remarkable physical attributes [14]. Superconductivity relies on the coherence length, and the electronic and magnetic properties of a superconductor undergo changes when its size is comparable to the coherence length [15,16]. Consequently, the diminution of size and dimensions of superconductors heralds novel insights into systematic exploration.

The allure of superconducting nanofibers lies in their potential to transcend conventional limitations and pave the way for innovative applications across diverse technological realms. The systematic manipulation of nanoscale dimensions holds the promise of unraveling nuanced behaviors and unlocking unprecedented functionalities in superconducting materials.

In essence, the pursuit of enhanced superconductivity encompasses a multifaceted quest spanning from fundamental investigations into the intricacies of material properties to the translation of these insights into practical advancements. As the landscape of superconductivity continues to evolve, the exploration of nanofibers and other nanostructured materials promises to chart new frontiers and redefine the boundaries of what is achievable in the realm of superconducting technologies. The superconducting properties of nanosized cuprate high-Tc superconductors (HTSCs) have garnered widespread attention, primarily due to the size effect altering the magnetic and electronic properties of superconductors, thereby offering a new perspective on unraveling the fundamental physics of superconductivity. Furthermore, the size dependence of room-temperature ferromagnetism, magnetic properties [17], and superconducting performance in HTSCs [18], as well as the coexistence of low-temperature ferromagnetism and superconductivity [19], have been investigated. These studies have yielded fresh insights into the fundamental characteristics of superconductors when scaled down in size.

Electrospinning, owing to its high efficiency, convenience, and excellent reproducibility, has emerged as one of the simplest and most viable methods for the production of superconducting nanofibers [20,21,22]. The cuprate superconducting nanofibers reported herein are prepared via electrospinning [23,24,25], followed by calcination, and the fiber morphology showed a uniform grain distribution, which increased the limit of cuprate to the nanometer level [16,20,21].

High-temperature superconducting materials exhibit numerous structural commonalities, which can be aptly elucidated using a laminar model. This model comprises a set of perovskite-type CuO_2_ atomic layers (known as copper oxide planes), serving as the foundational structural unit common to high-temperature superconducting materials, where superconductivity arises predominantly along these electrically conductive copper oxide planes. The copper oxide layers are sandwiched between two insulating structural layers, forming a laminar structure. These insulating layers are referred to as charge reservoir layers, and the genesis of superconductivity hinges upon the charge reservoir layers furnishing the requisite carrier concentration to the CuO_2_ planes. The lamellar CuO_2_ material constitutes the core structural element underlying its superconducting properties [22,23]. Consequently, if Sr^2+^ is infused into the La_2_CuO_4_ matrix instead of La^3+^, hole-type carriers will appear in the layer structure, which can achieve the effect of conduction between copper oxide surfaces. Additionally, when Sr is substituted for La, surplus holes migrate to oxygen sites within the copper oxide planes, forming O^−^ ions. The spins of oxygen hole states (O^−^) become strongly coupled to the spins of neighboring Cu^2+^ ions, inducing a ferromagnetic alignment of adjacent Cu^2+^ ions, thereby disrupting the long-range antiferromagnetic order. Low-energy neutron scattering studies reveal a robust correlation between the transition temperature (T_c_) and antiferromagnetic fluctuations within a certain doping range, underscoring the intricate interplay between them [24]. Generally, in La_2−x_Sr_x_CuO_4_, with the doping of impurities, the carrier concentration increases continuously and copper oxide undergoes an insulator–superconductor–normal metal transition; the optimal superconductor doped region is observed when x = 0.15 [25].

In this paper, we successfully employed electrospinning technology to prepare and characterize spider-web-like LSCO nanofibers. Our study revealed that under low temperatures, LSCO nanofibers exhibit superconducting phenomena, alongside the discovery of ferromagnetic responses.

## 2. Materials and Methods

### 2.1. Materials

To prepare LSCO superconducting nanofibers, lanthanum acetate (La(Ac)_3_·3H_2_O) (Energy Chemical Co., Ltd., Anhui, China), strontium acetate (Sr(Ac)_2_·5H_2_O)(Energy Chemical Co., Ltd., Anhui, China), and copper acetate (Cu(Ac)_2_·H_2_O) (Sinopharm Chemical Reagent Co., Ltd., Shanghai, China) were used as raw materials, which were, respectively, purchased from Energy Chemical Reagent Network and Sinopharm Chemical Reagent Group. Deionized water and polyvinyl alcohol (PVA) (Aladdin Scientific Corp., Shanghai, China) were purchased from the official website of Aladdin Chemical Reagents.

### 2.2. Material Preparation

To prepare the LSCO spinning precursor, the following procedure was followed: Specifically, 1.369 g of lanthanum acetate trihydrate, 0.065 g of strontium acetate and 0.3995 g of copper acetate were meticulously weighed and combined with 15 g of deionized water in a suitable container. The mixture was then stirred at room temperature for an extensive period of 2 h, allowing sufficient time for the compounds to completely dissolve and form a homogeneous solution.

Following the dissolution process, 1.5 g of polyvinyl alcohol (PVA) with a molecular weight of 66,000 was carefully added to the solution, which was subsequently stirred at a controlled temperature of 60 °C for a duration of 5 h. During this time, the solution gradually transformed into a distinctive sky-blue transparent gel state, indicating the formation of the precursor solution suitable for spinning.

Before proceeding with the spinning process, it was imperative to ensure the removal of any trapped air bubbles within the solution. Therefore, the solution was left to stand undisturbed for an additional 5 h, allowing for the complete discharge of air bubbles and ensuring the stability of the precursor solution.

The electrospinning apparatus used in this process comprised essential components including a high-voltage power supply, a collection instrument, a syringe, and a propulsion pump. To initiate the electrospinning process, an 18 kV high-voltage power supply was carefully connected to the needle, which served as the point of ejection for the precursor solution. Meanwhile, an aluminum foil-wrapped drum was employed as the collection device to capture the resulting nanofibers.

The distance between the needle and the collection device was meticulously set at 15 cm to optimize the spinning process and ensure the uniform deposition of nanofibers. Additionally, the feeding rate of the pump was precisely adjusted to 13 µL min^−1^, providing a controlled flow of the precursor solution during the electrospinning process. These meticulous adjustments were essential to achieve the desired quality and characteristics of the spun nanofibers for subsequent applications.

### 2.3. Characterization 

Scanning electron microscopy (SEM; Hitachi S-520, Hitachi High-Tech Co., Ltd., Tokyo, Japan) and transmission electron microscopy (TEM; Tecnai G2F20, Field Electron and Ion Company, Hillsboro, OR, USA) were used to observe the surface morphology and microstructure, respectively. Energy-dispersive X-ray spectroscopy (EDS; Hitachi S4800, Hitachi High-Tech Co., Ltd., Tokyo, Japan) was used to measure the elemental composition of LSCO nanofibers after calcination. The thermal stability of the composite fibers was analyzed using a thermogravimetric analyzer (TGA; Netzsch TG-209-F3, NETZSCH-Gertebau GmbH, Bavaria, Germany), and elemental peaks were characterized using an X-ray diffractometer (XRD; Rigaku D/MAX-2400, Rigaku Corporation, Tokyo, Japan). The magnetic properties of samples under mild and high magnetic fields were measured using a physical property measurement system (PPMS; Quantum Design Inc., California, CA, USA) with a vibrating sample magnetometer (VSM) module.

## 3. Results and Discussion

### 3.1. Thermogravimetric Analysis

**Figure 1:**
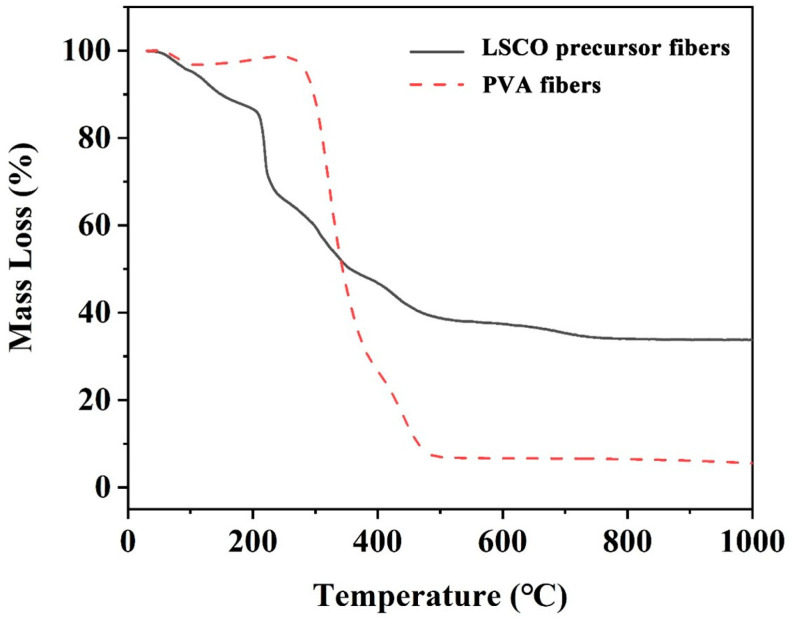
Thermogravimetric analysis of electrospun La_1.8_5Sr_0.15_CuO_4_ (LSCO) precursor fibers and pure polyvinyl alcohol (PVA) fibers.

Figure 1 illustrates the thermal behavior analysis conducted on LSCO/PVA composite nanofibers utilizing Thermogravimetric Analysis (TGA). The LSCO/PVA composite nanofibers depicted a distinctive softening and melting behavior around 150 °C. As the temperature surpassed 250 °C, a substantial degradation of PVA occurred, attributed to its decomposition into acetic acid, acetaldehyde, butenol, and water, with the degradation rate escalating with temperature. Upon reaching 800 °C, the mass loss exceeded 90%, signifying the complete decomposition of the nanofiber product into CO_2_ and H_2_O.

The thermal decomposition process of LSCO/PVA composite nanofibers unfolded through three discernible stages. Initially, below 200 °C, a gradual decline in mass was observed owing to the evaporation of water content within the composite fibers. Subsequently, as the temperature ascended, the rapid degradation of PVA induced a substantial decline in overall nanofiber quality. By the time the temperature soared to 700 °C, the mass alteration trend plateaued gradually, marking the complete volatilization of organic constituents and the onset of LSCO grain growth.

Based on insights gleaned from the thermogravimetric analysis profile, a systematic combustion regimen was devised to procure single-phase LSCO. Initially, LSCO composite fibers were subjected to heating in an air atmosphere until the temperature reached 230 °C, with a heating rate of 3 °C min^−1^, and maintained for 1 h. This facilitated rapid moisture evaporation from the nanofibers and the softening and melting of organic constituents. Subsequently, the heating rate was sustained at 3 °C min^−1^ until reaching 450 °C, where the temperature was sustained for 1 h, enabling complete decomposition of organic constituents within the composite fibers. To promote optimal grain growth, the composite fibers underwent further heating to 700 °C for a duration of 3 h, maintaining the same heating rate.

Considering LSCO’s susceptibility to oxygen deprivation, the resultant sintered product underwent gradual cooling to 500 °C and underwent annealing in a high-purity oxygen environment, sustained at a flow rate of 50 cm^3^ min^−1^. Subsequently, the sample was allowed to cool naturally to ambient temperature, ensuring the preservation of its structural integrity and crystalline purity.

This comprehensive thermal analysis coupled with meticulous combustion procedures not only elucidates the intricate decomposition behaviors of PVA and LSCO composite nanofibers but also delineates a systematic approach to fabricating single-phase LSCO with enhanced structural properties and compositional purity, thus underpinning its applicability across diverse technological domains.

### 3.2. XRD

**Figure 2:**
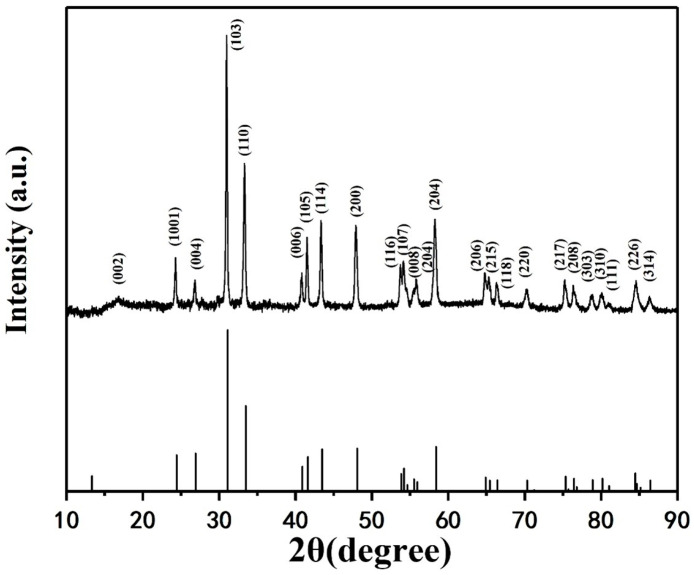
XRD patterns of LSCO nanofibers after calcination.

The X-ray diffraction (XRD) analysis conducted on LSCO superconducting nanofibers provided comprehensive insights into their phase structure. Figure 2 illustrates the XRD pattern obtained from the calcined LSCO nanofibers, revealing distinctive reflection peaks that unequivocally correspond to the single-phase tetragonal LSCO (JCPDS 79-0456). Notably, the absence of secondary oxide phases underscores the purity of the synthesized nanofibers. The observed reflection peaks exhibit pronounced intensity and narrow peak widths, indicative of superior crystallinity and lattice integrity within the calcined LSCO nanofibers.

The determination of grain size, crucial for understanding nanoscale properties, was accomplished employing the Debye–Scherrer equation:

(1) $$D=\frac{K\gamma }{\mathrm{Bcos}\theta }$$

Herein, *D* represents the average thickness of grains oriented orthogonally to the crystal plane direction, denoting the grain size. The Scherrer constant *K* is established as 0.89. The parameter B signifies the integral width or half-height width of the diffraction peak, as obtained from the experimental sample, while *θ* denotes the Bragg angle utilized in the XRD analysis. Moreover, *γ* denotes the X-ray wavelength, with a specific wavelength of 0.154056 nm utilized in this investigation. Applying the aforementioned parameters, the average grain size of the LSCO nanofibers was determined to be 65.65 nm. This calculated grain size offers valuable insights into the nanoscale morphology and structural characteristics of the LSCO nanofibers.

The XRD characterization of LSCO superconducting nanofibers demonstrates the predominant presence of tetragonal LSCO phase, free from secondary oxide phases, highlighting the purity and structural integrity of the synthesized nanofibers. Furthermore, the calculated average grain size elucidates the nanoscale dimensions of the LSCO nanofibers.

### 3.3. SEM and TEM

**Figure 3:**
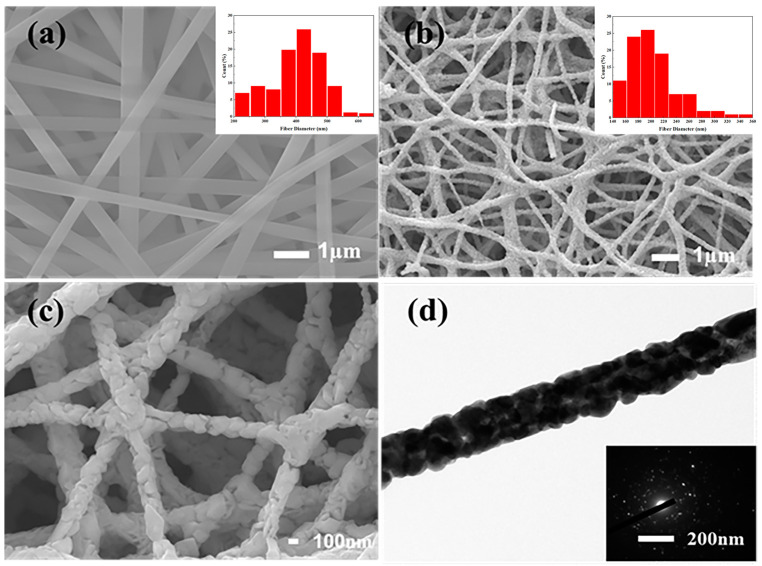
SEM and TEM images of LSCO nanofibers before and after calcination. (**a**) Fiber morphology and diameter analysis of LSCO nanofibers before calcination. (**b**,**c**) Fiber morphology and diameter analysis of LSCO nanofibers after calcination. (**d**) Transmission image and polycrystalline diffraction pattern of LSCO nanofibers after calcination.

The morphological evolution of LSCO nanofibers, crucial for understanding their structural integrity and functional properties, is meticulously elucidated through a comprehensive analysis employing scanning electron microscopy (SEM) and transmission electron microscopy (TEM), both pre- and post-sintering processes, as depicted in Figure 3.

Figure 3a offers a nuanced depiction of the pristine morphology and diameter distribution of LSCO/PVA precursor nanofibers preceding calcination. Electrospun nanofibers, characterized by their uniform diameter and sleek surface topology, epitomize the precision afforded by the electrospinning process. The observed diameter distribution, centered at 450 ± 50 nm, reflects the inherent homogeneity within the electrospun fibers, albeit punctuated by sporadic deviations attributable to transient instabilities encountered during the electrospinning process.

Subsequent to the transformative crucible of sintering, Figure 3b,c unravel the metamorphosis of LSCO nanofibers across varying magnifications, elucidating the intricacies of structural refinement engendered by thermal processing. The calcination process engenders a discernible reduction in nanofiber diameter to 200 ± 50 nm, underscoring the efficacy of thermal treatments in PVA residues and facilitating the crystallization of LSCO, thereby endowing the fibers with enhanced structural integrity and crystalline coherence. Notably, the preservation of fibrous morphology amidst thermal vicissitudes attests to the robustness of the electrospun LSCO nanofibers, thereby reinforcing their suitability for diverse applications spanning the domain of advanced materials science.

The nanoarchitectural underpinnings of LSCO nanofibers emerge as a interplay between nanograins and nanopores intricately woven within the fiber matrix. The amalgamation of 65.65 nm LSCO particles into one-dimensional superconducting wires underscores the profound influence of nanoscale confinement on the emergent properties of materials, culminating in the formation of a porous surface morphology conducive to interfacial interactions and structural synergies. Throughout the grain growth process, nanofibers assume a porous configuration, interlinking the internal structural elements to fabricate a spider-web-like network of nanowires, thereby endowing the surface with a myriad of pores of varying dimensions, thereby conferring multifaceted functionalities to the LSCO nanofiber ensemble.

In juxtaposition to conventional templating methodologies, the electrospinning technique affords unparalleled advantages in the synthesis of superconducting nanofibers. Unlike templating methods constrained by template thickness, electrospun nanowires boast extended lengths of up to 100 μm, facilitating the creation of a contiguous fiber network conducive to sustained current conduction. The spider-web-like network structure, characteristic of electrospun nanofibers, engenders numerous interconnection points, thereby augmenting the pathways available for current propagation, thus underscoring the superior potential of electrospinning in the fabrication of nanofiber superconducting materials.

Figure 3d unveils a TEM image of a solitary nanowire in dark field, encapsulating the essence of crystalline coherence and structural fidelity exhibited by the calcined fibers. The diffraction pattern, characterized by polycrystalline diffraction spots and diffraction rings, underscores the rich tapestry of structural complexity underpinning LSCO nanofibers, thereby accentuating their promise as paradigm-shifting constituents of next-generation materials platforms.

### 3.4. EDS

**Figure 4:**
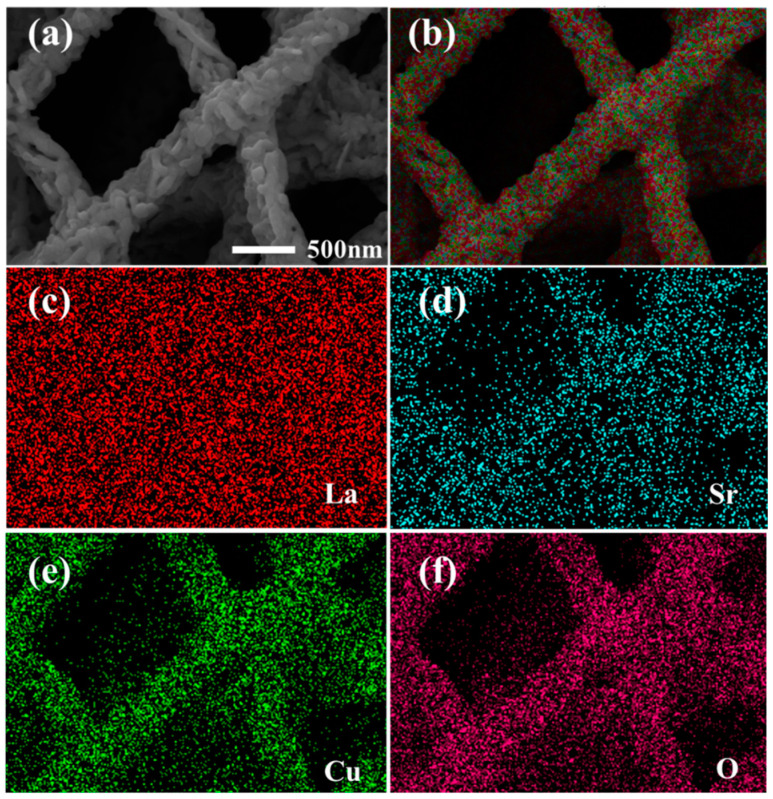
(**a**) LSCO nanofibers after calcination. (**b**) Overall element distribution. (**c**–**f**) Distribution of La, Sr, Cu, and O in the nanofibers.

In order to meticulously ascertain the elemental composition of the calcined LSCO nanofibers, energy-dispersive X-ray spectroscopy (EDS) analysis was meticulously conducted. Figure 4a presents a microscopic portrayal of the nanofibers, while Figure 4b–f intricately delineate the uniform distribution of La, Sr, Cu, and O elements across the nanofibrous ensemble.

The discernible uniformity in elemental distribution underscores the meticulous precision afforded by the fabrication process, facilitating the homogeneous integration of constituent elements essential for the structural and functional integrity of LSCO nanofibers. The pronounced uniformity in elemental distribution not only corroborates the fidelity of the synthesis methodology but also augments our confidence in the structural coherence and compositional fidelity of the fabricated nanofibers.

Moreover, the meticulously executed synthesis and calcination protocols preclude the likelihood of oxygen deficiency within the synthesized nanofibers. The absence of oxygen deficiency, both during the calcination process and subsequent oxygen flow annealing, attests to the meticulous control exercised over the synthesis parameters, ensuring the preservation of stoichiometric balance essential for the manifestation of desired structural and functional attributes within the LSCO nanofiber ensemble.

### 3.5. Magnetic Analysis

**Figure 5:**
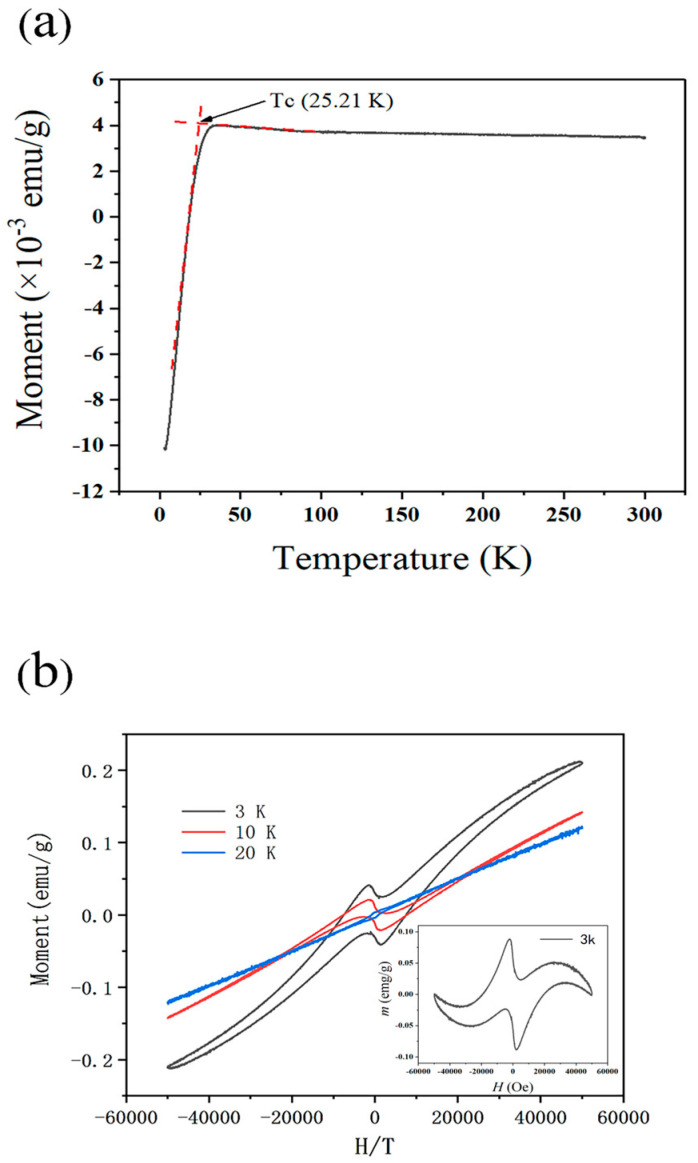
(**a**) Magnetic zero-field-cooled (ZFC) M-T curve under a 0.005 T field. The red dashed lines are used to extract the onset temperature. (**b**) Magnetic hysteresis loops of LSCO at different temperatures; the inset displays the magnetic hysteresis loop curve without the magnetic background subtracted at 3 K.

The PPMS was utilized to conduct tests, along with the zero-field-cooled (ZFC) method. Figure 5a shows the variation in magnetization with temperature and the background magnetic field was 0.005 T. Near the critical superconducting temperature, Tc, at approximately 25.21 K, a rapid decline in magnetization is observed in the sample. The broad transition width of the sample reflects its granular structure and potential structural non-uniformity, indicating slight variations in reaction across different regions of the sample at the same temperature. However, this broadening may also stem from the presence of a ferromagnetic phase within the system [18]. This broadening could also arise due to the interaction of magnetic domains within the sample, causing a gradual alignment of spins leading to a more gradual transition in magnetization. Furthermore, the presence of impurities or defects at grain boundaries might contribute to the observed broadening phenomenon, affecting the overall magnetic response of the material. These factors suggest a complex interplay of magnetic interactions within the sample, warranting further investigation into its microstructure and composition. 

Despite measurements at the lowest temperature (3 K), the sample still does not exhibit complete flux exclusion. This is attributed to the sample’s diameter nearing the magnetic penetration depth (typically a few hundred nanometers), rendering it challenging for nanoscale superconducting materials to achieve complete flux exclusion [26]. Interestingly, in previous studies related to electrospun LSCO nanofibers, their Tc values were reported as 19.2 K [27] and 29.3 K [28], respectively. This variation in Tc is likely correlated with the particle size of LSCO nanograins. Specifically, the Tc values of 19.2 K and 29.3 K for electrospun LSCO nanofibers correspond to average grain sizes of 18.4 nm and 110 nm, respectively. In this current study, the Tc value of 25.21 K and the average grain size D = 65.65 nm lie between these two extremes. This suggests that the decrease in T_c_ is somewhat related to the reduction in grain diameter within the LSCO nanofibers. This relationship is further corroborated by a study investigating the correlation between LSCO nanoparticle size and superconducting performance [24].

The decrease in T_c_ with decreasing particle size can be attributed to several factors. Firstly, the London penetration depth, which is comparable to the particle size, will reduce superconducting diamagnetism. Secondly, uncompensated spins from surface effects may induce ferromagnetic and/or paramagnetic behavior, enhancing the positive contribution to magnetization. The presence of surface ferromagnetism and paramagnetism further inhibits the tendency toward superconducting order, thus lowering the critical temperature. Additionally, in smaller particles, the role of surface defects (such as oxygen vacancies) becomes increasingly significant and may weaken superconductivity. Similarly, these defects may lead to the development of ferromagnetic moments within surface atoms, which could suppress the development of the superconducting phase caused by the pair-breaking effect [18]. This is consistent with the observed trend of decreasing T_c_ in smaller-sized particles, as smaller particles also exhibit the strongest ferromagnetism.

Figure 5b displays the magnetization curves of LSCO nanofibers at temperatures of 3, 10, and 20 K. The Fishtail effect [29] was evident and specifically characterized by conspicuous peaks that emerged under diminished magnetic field intensity. This is indicative of the typical flux pinning effect observed in type II superconductors. Additionally, it is notable that the magnetization curve transitions into a region of positive magnetization with increasing applied magnetic field, exhibiting a large positive slope and noticeable curvature, typical characteristics of a ferromagnetic phase, as clearly observed in Figure 5b inset. As temperature increases, the contribution of superconductivity to the curve gradually diminishes. At 20 K, the curve demonstrates a distinct hysteresis loop characteristic of ferromagnetic behavior. Furthermore, with increasing temperature, the coercive force, magnetization intensity, and remanence of the ferromagnetic phase all decrease.

The emergence of this ferromagnetism could be explained by several possibilities. In GbBaCuO nanobelts, magnetization curves at low temperatures exhibit unexpectedly broadened vortex profiles, which are believed to result from nanoscale pinning sites causing the broadening of vortex profiles and the generation of magnetization curves [30]. Similarly, impurities at copper positions in bulk copper-based superconductors create pinning sites that lead to the weakening of short-range magnetic order and superconductivity [31,32,33]. Nanoscale superconducting particles may also generate magnetization due to these effects.

Another possible explanation originates from charge redistribution of atoms near the surface [34,35], affecting the bonding between copper and adjacent oxygen molecules (such as Cu1-O1 and Cu1-O2). Surface-related defects often form an impurity band, which mixes with the redistributed 3d electron states of copper, leading to an abundance of uncompensated spins on the surface. Since the copper-O-copper superexchange interaction is expected to be a strong function of bond length, larger surface-to-volume ratios increase the concentration of oxygen defects on the surface, leading to more disruption of copper-O-copper interactions and introducing additional uncompensated surface spins. These uncompensated spins may couple through direct or indirect interactions, resulting in weak ferromagnetism [19].

Another noteworthy phenomenon observed in the magnetization curves of this study is the simultaneous presence of ferromagnetic and superconducting phases at a low temperature of 3 K. This is quite uncommon in LSCO nanoscale superconductors. For instance, in LSCO nanograins, the superconducting behavior completely disappears when the size is reduced to 66 nm, transitioning to Curie behavior [36]. Although the particle size of LSCO nanofibers in this study is 65.56 nm, this phenomenon is not observed. Instead, a pronounced coexistence of ferromagnetism and superconductivity is evident. The cause of this phenomenon may be attributed to the interconnection and sintering of grains in LSCO nanofibers. In comparison to nanograins, LSCO nanofibers have a lower proportion of surface (ferromagnetic) to core (superconducting) phases, resulting in different magnetic behaviors. This observation provides insight into the question of whether ferromagnetic and superconducting phases coexist in the nanostructure.

## 4. Conclusions

In the pursuit of advancing superconducting materials, we have achieved a significant milestone through the fabrication of cobweb-like La_2-x_Sr_x_CuO_4_ superconducting nanofibers via an intricate process involving electrospinning coupled with multistep calcination. The resultant nanofibers exhibit intriguing superconducting properties, elucidated through meticulous analyses of magnetic images encompassing zero-field-cooled (ZFC) magnetization–temperature (M–T) curves, alongside the scrutiny of hysteresis loops. Notably, our investigation reveals a critical transition temperature T_c_ of 25.12 K for the LSCO nanofibers, a value that manifests discernible disparities from extant literature. This variance in T_c_ is primarily attributed to the divergent sizes of the superconducting nanofibers, underscoring the established correlation wherein smaller particle dimensions engender diminished T_c_ values.

Furthermore, our scrutiny unveils a coalescence of ferromagnetic and superconducting phases within the sample below the temperature of 20 K, a phenomenon accentuated with declining thermal conditions. We ascribe this intriguing juxtaposition to the overarching influence of nanoscale size effects, positing that an augmented prevalence of surface states precipitates the emergence of ferromagnetism. A nuanced comprehension of these size-dependent phenomena assumes paramount significance in the iterative refinement and optimization of nanostructured superconductors vis-a-vis their pragmatic deployment in diverse applications. This seminal discovery not only augments the pantheon of knowledge concerning the magnetic intricacies inherent to electrospun LSCO nanofibers but also furnishes a fresh vantage point for the proliferation of LSCO nanofiber applications, thereby catalyzing further explorations into the domain of high-temperature superconductors.

To unravel the underlying mechanisms in greater detail, it behooves us to embark on a systematic exploration delineating the repercussions of distinct particle dimensions of electrospun LSCO nanofibers on magnetism. Additionally, employing advanced electron microscopy techniques holds promise in elucidating the intricacies of surface and internal vortex profiles within LSCO, thereby furnishing invaluable insights into the underlying physics governing these materials.

In conclusion, the synthesis and characterization of cobweb-like LSCO superconducting nanofibers represent a seminal stride towards unraveling the enigmatic tapestry of high-temperature superconductors. Through judicious experimentation and rigorous analysis, we endeavor to illuminate the intricate interplay between size-dependent phenomena and material properties, thereby charting a course towards the realization of next-generation superconducting technologies with unparalleled efficacy and versatility.

## Data Availability

The original contributions presented in the study are included in the article/supplementary material, further inquiries can be directed to the corresponding author/s.
